# RACK1 depletion in the ribosome induces selective translation for non-canonical autophagy

**DOI:** 10.1038/cddis.2017.204

**Published:** 2017-05-18

**Authors:** Hag Dong Kim, EunBin Kong, YongJoong Kim, Jin-Soo Chang, Joon Kim

**Affiliations:** 1 Laboratory of Biochemistry, Division of Life Sciences, Korea University, Seoul 02841, Republic of Korea; 2 HAEL, TechnoComplex Building 603-3, Korea University, Seoul 02841, Republic of Korea

## Abstract

RACK1, which was first demonstrated as a substrate of PKC*β* II, functions as a scaffold protein and associates with the 40S small ribosomal subunit. According to previous reports, ribosomal RACK1 was also suggested to control translation depending on the status in translating ribosome. We here show that RACK1 knockdown induces autophagy independent of upstream canonical factors such as Beclin1, Atg7 and Atg5/12 conjugates. We further report that RACK1 knockdown induces the association of mRNAs of LC3 and Bcl-xL with polysomes, indicating increased translation of these proteins. Therefore, we propose that the RACK1 depletion-induced autophagy is distinct from canonical autophagy. Finally, we confirm that cells expressing mutant RACK1 (RACK1^R36D/K38E^) defective in ribosome binding showed the same result as RACK1-knockdown cells. Altogether, our data clearly show that the depletion of ribosomal RACK1 alters the capacity of the ribosome to translate specific mRNAs, resulting in selective translation of mRNAs of genes for non-canonical autophagy induction.

RACK1 (receptor for activated C-kinase 1) is a scaffold protein that can bind hundreds of other proteins. RACK1 was first identified as a signaling molecule that interacts with PKC*β*II to contribute to cellular growth, movement and differentiation.^1, 2, 3^ Because it lacks catalytic domains,^4^ RACK1 functions as a molecular ship that shuttles activated PKC*β*II and various other signaling proteins, including integrin and Src. The interaction between RACK1 and various proteins in different cellular compartments plays a critical role in many physiological processes, such as cell motility, cell survival and death, proliferation, immune signaling, tumorigenesis and neuronal function.^5, 6, 7, 8, 9, 10^ Also, RACK1 can regulate global and specific translations in different ways. RACK1 is an integral component of ribosomal 40S subunit,^11^ acts as a signaling platform for the translational machinery and regulates a late step in translation initiation.^12, 13^ Ribosomal RACK1 is located at the head region of the 40S subunit, in the vicinity of the mRNA exit channel.^14^ When a cell receives a signal that promotes translation, RACK1-associated PKC*β*II phosphorylates eIF6. In succession, phosphorylated eIF6 is released from the 60S subunit to induce 80S ribosome formation.^15, 16^ Furthermore, ribosomal RACK1 is required for IRES-mediated translation after viral infection,^17^ suggesting a function for ribosomal RACK1 in selective mRNA translation. Deletion of the yeast RACK1 homolog Asc1 is not lethal,^18^ whereas the complete depletion of RACK1 in mouse embryo is lethal. Moreover, *Rack1* hypomorphic mice are deficient in protein synthesis.^19^ Also, in nematodes and mammals, RACK1 contributes to the recruitment of miRISC (miRNA-induced silencing complex) to the ribosome complex, followed by miRNA-mediated regulation of gene expression at the post-transcriptional level.^20^ However few reports have addressed the relationship between RACK1 and autophagy.^21^

Cells strive to maintain beneficial environmental conditions. This self-adjusting mechanism is called homeostasis. Eukaryotic cells have two protein degradation processes to maintain cellular homeostasis; one is an ubiquitin–proteasome system (UPS) and the other is an autophagy.^22^ In contrast to UPS, autophagy selectively eliminates protein aggregates and useless cellular organelles. In general, autophagy is a slower mechanism than the UPS and it degrades relatively long-lived proteins. Autophagy is classified into two categories according to the target. One is selective autophagy and the other is non-selective autophagy. Non-selective autophagy is involved in the random degradation and sequestration of a portion in a cytosol. However, selective autophagy is more complex. It can degrade specific protein aggregates or organelles using adaptor proteins such as p62/SQSTM1 and NBR1.^23^ Ribophagy, mitophagy and reticulophagy are examples of selective autophagy.^24, 25^ In autophagy signaling pathways, the two types of autophagy have much in common. Association of LC3 with a double-membrane structure, phagophore, is a key step in the autophagic mechanism. The phagophore is extended by additional LC3, engulfs the target protein and forms an autophagosome, which fuses with lysosomes to form autophagolysosomes, resulting in lysosome-mediated degradation. Thus, components of proteins or cellular organelles are reused for adaptation to new environments.^26^

Autophagy is a complex process controlled by numerous autophagy-related (Atg) proteins.^27, 28^ Under nutrient-rich conditions, mTOR complex 1 suppresses the ULK1 complex. Upon autophagy induction, the ULK1 complex is activated, which is the first step in canonical autophagy.^29^ Active ULK1 complex induces the class III phosphatidylinositol (PtdIns) 3-kinase complex (Beclin1 complex) and mediates the initial stages of vesicle nucleation, resulting in autophagosome formation.^30^ Meanwhile, Beclin1 forms a complex with Bcl-2 or Bcl-xL under normal conditions, which inhibits canonical autophagy. The interaction is disrupted by JNK1-mediated Bcl-2 or Bcl-xL phosphorylation, which is followed by autophagy induction.^31, 32^ After nucleation by the Beclin1 complex, two ubiquitin-like conjugation systems, the Atg5–Atg12 system and the LC3 conjugation system, cause autophagosomal elongation.^33^ The last step of canonical autophagy is autophagolysosome formation by the fusion between autophagosomes and lysosomes.^34, 35^ Several other autophagy mechanisms, which are now referred to as a non-canonical autophagy, are not related to the canonical autophagy pathway.^36^ An example of non-canonical autophagy is Beclin1-independent autophagy, which does not require the canonical nucleation step mediated by the Beclin1 complex.^37^ However, the molecular mechanism of non-canonical autophagy is unclear. Our data suggested that RACK1 depletion stimulates non-canonical autophagy due to induction of LC3 and Bcl-xL translation.

## Results

### Knockdown of RACK1 induces autophagy in HT1080 cells

**Figure 1:**
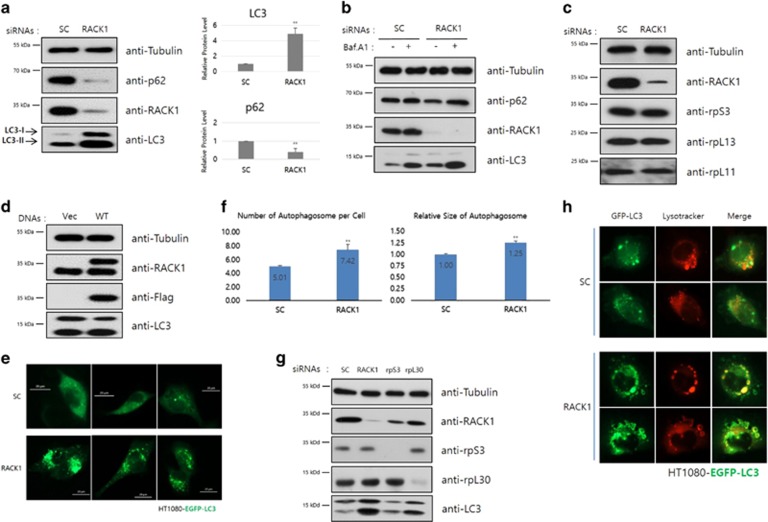
(**a**) HT1080 cells were transfected with control or RACK1 siRNAs (50 pmol), and then incubated for 48 h. The cell lysates were subjected to immunoblot analysis using the indicated antibodies. LC3-II and p62/SQSTM1 are markers of autophagic flux (left panel). Intensities of LC3 and p62 proteins were normalized against that of tubulin proteins, and the relative expressions in RACK1 siRNA-treated cells compared with that in control cells was plotted (right panel). **P<0.01 (Student’s *t*-test). (**b**) Extracts of siRNA-transfected cells were pretreated with Bafilomycin A1 (1 *μ*M) for 1 h, and subjected to immunoblot analysis using the indicated antibodies. (**c**) Each siRNA-treated HT1080 cells were lysed and extracts were subjected to immunoblot analysis using the indicated antibodies. (**d**) Immunoblot analysis using the indicated antibodies was performed after transient transfection of plasmids, pcDNA3-Flag vector or pcDNA3-Flag-RACK1 (WT) into HT1080 cells. (**e** and **g**) Indicated siRNAs (50 pmol) were transfected into EGFP-LC3 stable HT1080 cells. GFP-LC3 dots were detected by fluorescence microscopy (**e**), and the cell lysates were analyzed by immunoblotting using the indicated antibodies (**g**). (**f**) HT1080 cells were transfected with control or RACK1 siRNAs (50 pmol), CYTO-ID Green Detection Reagent is added, and then detected and analyzed. **P<0.01 (Student’s *t*-test). (**h**) After 48 h, GFP-LC3 stable HT1080 cells were treated with control or RACK1 siRNA (50 pmol), and then incubated with 50 nM LysoTracker for 30 min. After washing with PBS, images were obtained by florescence microscopy. The merged yellow signals of LysoTracker (red) and GFP-LC3 (green) indicated the autophagolysosome, which is formed by fusion of the lysosome and autophagosome

RACK1 participates in numerous cellular functions through interacting with many signaling proteins. We here showed the activation of autophagy when RACK1 siRNA is treated, judging from the levels of two autophagic markers, p62 and LC3.^38, 39^ The level of p62/SQSTM1, an autophagy receptor, was reduced and that of LC3-II was increased by RACK1 knockdown (Figure 1a). Subsequently, we treated control and RACK1-knockdown cells with Bafilomycin A1, an autophagy inhibitor that blocks the fusion of autophagosome and lysosome. As shown in Figure 1b, p62/SQSTM1 was restored in Bafilomycin A1-treated RACK1-knockdown cells. We demonstrated that this phenomenon was not caused by an siRNA off-target effect. Other siRNAs that target different regions of the RACK1 mRNA sequence also induced autophagy (Supplementary Figures S1A and C). According to previous reports in yeasts, nonfunctional or abnormal ribosomes were shown to induce a selective autophagy known as ribophagy, which degrades the ribosome complex. Therefore, we checked the levels of other ribosomal proteins that belong to both 40S small and 60S large subunits. The rpS3, rpL13 and rpL11 protein levels were little changed by RACK1 knockdown (Figure 1c). Next, as RACK1 is a scaffold protein involved in numerous signaling pathways, we assessed its participation in autophagy signaling. However, overexpression of RACK1 did not affect the level of LC3 in HT1080 cells (Figure 1d). Thus, RACK1 does not seem to be an inhibitor of autophagy.

To extend our finding of RACK1-knockdown-induced autophagy, we investigated the accumulation of GFP-LC3 puncta by fluorescence microscopy. We generated a stable cell line expressing EGFP-LC3, and then the cells were treated with siRNAs for control and RACK1. The number of EGFP-LC3 puncta and relative size of autophagosome were markedly increased in RACK1-knockdown cells compared with control (Figures 1e and f). In addition, the LC3-II protein level and the number of EGFP-LC3 puncta are significantly higher only in RACK1-knockdown cells compared with rpS3- and rpL30-knockdown cells (Figures 1g and Supplementary Figure S1D). In addition, in the stable cell line, co-localization of the autophagosome and lysosome was examined.^40^ RACK1-knockdown cells exhibited significantly elevated co-localization level of GFP-LC3 puncta and LysoTracker, compared with control cells (Figure 1h). These data collectively indicate that RACK1 knockdown induces autophagic flux in terms of an increment in p62/SQSTM autophagic degradation and autophagolysosome formation.

### RACK1 depletion induces non-canonical autophagy

**Figure 2:**
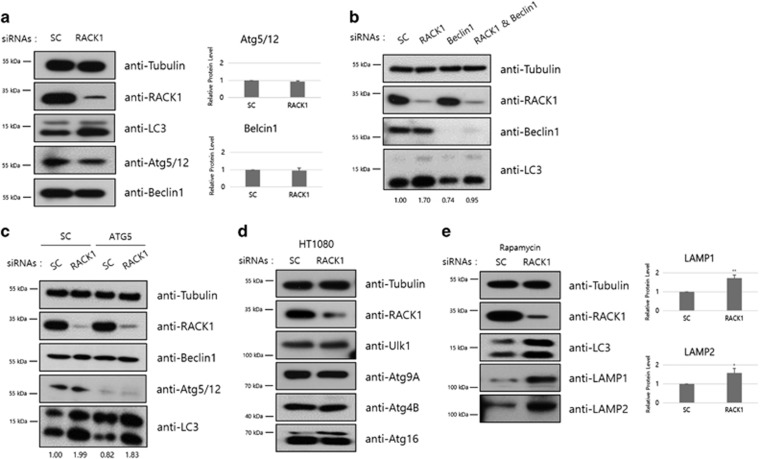
RACK1 depletion-induced autophagy is non-canonical. (**a**) HT1080 cells transfected with control or RACK1 siRNAs (50 pmol) were incubated for 48 h, followed by immunoblot analysis using the indicated antibodies (left panel). Intensities of Atg5/12 and Beclin1 proteins were normalized against that of tubulin proteins, and the relative expressions in RACK1 siRNA-treated cells compared with that in control cells were plotted (right panel). (**b**) HT1080 cells were transfected with siRNAs against control or RACK1 in combination with or without Beclin1 siRNA (100 pmol). After 48 h, these cells were analyzed by immunoblotting using the indicated antibodies. (**c**) HT1080 cells were transfected with control or Atg5 siRNA (100 pmol). After 24 h, the cells were re-transfected with control or RACK1 siRNA. After a further 48 h incubation, the cell extracts were subjected to immunoblot analysis using the indicated antibodies. (**d**) HT1080 cells transfected with control or RACK1 siRNAs (50 pmol) were incubated for 48 h, followed by immunoblot analysis using the indicated antibodies. (**e**) HT1080 cells transfected with control or RACK1 siRNAs (50 pmol) were incubated for 24 h. Extracts of siRNA-transfected cells were pretreated with rapamycin 1 μM for 24 h, followed by immunoblot analysis using the indicated antibodies. Intensities of LAMP1 and LAMP2 proteins were normalized against that of tubulin proteins, and the relative expressions in RACK1 siRNA-treated cells compared with that in control cells were plotted. *P<0.05, **P<0.01 (Student’s *t*-test)

Autophagy has a very complicated mechanism and a lot of factors that contribute to its flux in diverse ways. Pointing out a few examples, ULK1, Atg13 and RB1CC1 are major components of the Ulk1 complex, which is involved in the initiation step of autophagy^29^ and antiapoptotic proteins, Bcl-xL and Bcl-2, function as an inhibitor of Beclin1 complex.^32^ Among the intricate pathway, to characterize the RACK1-knockdown-induced autophagic flux in detail, we examined the pathways upstream and downstream of LC3. Especially, we checked the levels of two significant factors essential for canonical autophagy, Beclin1 and conjugated Atg5/12. Beclin1 is a mammalian homolog of yeast Atg6, which constitutes the class III PI3K complex.^30^ The class III PI3K complex contributes to an upstream step of autophagic flux such as vesicle nucleation. Conjugated Atg5/12 is involved in autophagosome membrane elongation. It is part of the ubiquitin-like conjugation system and functions as an E2-like enzyme for lipidation of LC3-I protein.^33^ As shown in Figure 2a, the levels of these proteins were not increased by treatment with RACK1 siRNA. Additionally, HT1080 cells were transfected with RACK1 siRNA together with Beclin1 siRNA (Figure 2b) or ATG5 siRNA (Figure 2c). Because Beclin1 and Atg5 are positive regulators of canonical autophagy, knockdown of Beclin1 and Atg5 resulted in a reduced level of LC3-II, indicating blockade of canonical autophagy. However, the increased LC3 protein level induced by RACK1 knockdown was not blocked by Beclin1 or Atg5 siRNA co-treatment. Also, we reconfirmed that RACK1 knockdown did not increase the levels of Beclin1 or Atg5/12 conjugates. Therefore, the increased level of LC3-II induced by RACK1 knockdown results in Beclin1 and Atg5/12-independent non-canonical autophagy.

Moreover, RACK1 depletion-induced autophagy was independent of the levels of several other autophagy-related factors (Figure 2d). When treated with RACK1 siRNA, the protein levels of Ulk1, Atg9a, Atg4b and Atg16 were unaffected. These proteins were increased under serum-starvation conditions as an example of canonical autophagy induction (Supplementary Figure S2B). Thus, the RACK1-knockdown-induced autophagy follows a non-canonical pathway. Interestingly, we detected a significant increment of LAMP1 and LAMP2 levels in RACK1-knockdown cells (Figure 2e). Lysosomal-associated membrane protein 1 and 2 (LAMP1 and LAMP2), lysosomal membrane glycoproteins,^41^ are downstream markers of autophagy that facilitate autophagolysosome formation. A similar increment was detected under serum-starvation conditions (Supplementary Figure 2C).

Given that the phosphorylation of Ulk1 at serine 758 by mTOR inhibits canonical autophagy, we next examined the phosphorylation status of Ulk1. We found that RACK1 knockdown does not affect the Ulk1 protein level, but induces an increment of the Ser758 phosphorylation status of Ulk1 (Supplementary Figure S2E). The increased phosphorylation on Ser758 of Ulk1 by RACK1 knockdown was even more clearly observed under starvation condition (Supplementary Figure S2F). These findings strongly suggested that RACK1 knockdown induces non-canonical autophagy that is independent of initiation step as indicated by the increment in LC3 protein.

### Knockdown of RACK1 increases Bcl-xL protein level

**Figure 3:**
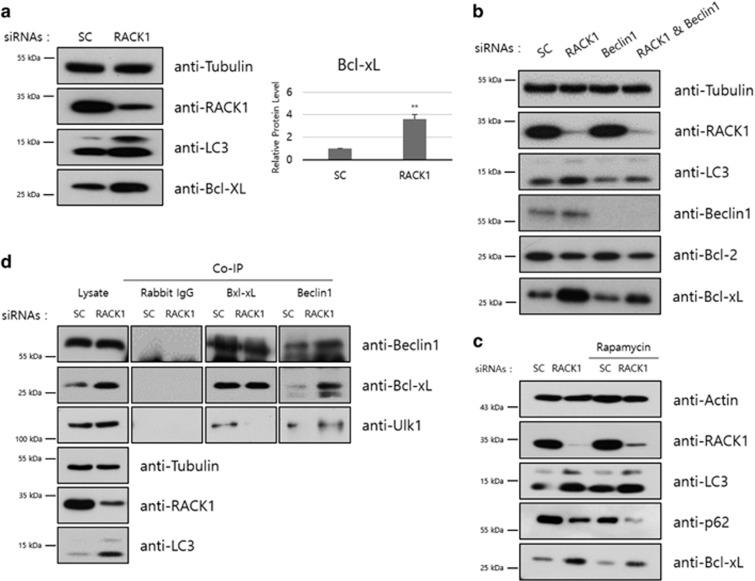
Knockdown of RACK1 increases Bcl-xL protein level. (**a**) HT1080 cells transfected with control or RACK1 siRNAs (50 pmol) were incubated for 48 h, followed by immunoblot analysis using the indicated antibodies (left panel). Intensities of Bcl-xL protein was normalized against that of tubulin protein, and the relative expression in RACK1 siRNA-treated cells compared with that in control cells was plotted (right panel). **P<0.01 (Student’s *t*-test). (**b**) HT1080 cells were transfected with siRNAs against control or RACK1 in combination with or without Beclin1 siRNA (100 pmol). After 48 h, these cells were analyzed by immunoblotting using the indicated antibodies. (**c**) HT1080 cells transfected with control or RACK1 siRNAs (50 pmol) were incubated for 48 h. Extracts of siRNA-transfected cells were pretreated with rapamycin 1 μM for 24 h, followed by immunoblot analysis using the indicated antibodies. (**d**) Extracts obtained from cells transfected with each siRNA (20 pmol) were immunoprecipitated using Bcl-xL and Beclin1 antibodies. Total cell lysates and the immunoprecipitates were resolved and subjected to immunoblot analysis using the indicated antibodies

In addition to these, we further found that the Bcl-xL level was significantly increased by treatment with RACK1 siRNA (Figure 3a). Bcl-xL is an antiapoptotic protein that contributes to cell survival and inhibits canonical autophagy by binding to the Beclin1 complex.^31, 32^ Then the increment of Bcl-xL was not affected by Beclin1 siRNA treatment (Figure 3b) and by rapamycin treatment (Figure 3c). Bcl-xL belongs to Bcl-2 protein family and Bcl-xL and Bcl-2 are very similar,^42^ but their expression patterns were quite different when RACK1 was depleted (Figure 3b). Because Bcl-xL and Beclin1 interact under basal conditions to regulate autophagy induction, we in turn performed co-IP experiment to examine the effect of RACK1 knockdown on the interaction (Figure 3d). The Bcl-xL and Beclin1 interaction was markedly increased in RACK1-knockdown cells as compared with control cells, indicating repression of canonical autophagy. These findings suggested that RACK1 knockdown induces non-canonical autophagy as indicated by the increments of LC3 protein and of the interaction of Beclin1 with Bcl-xL.

### RACK1-knockdown-induced autophagy is not a cell-line-specific phenomenon

**Figure 4:**
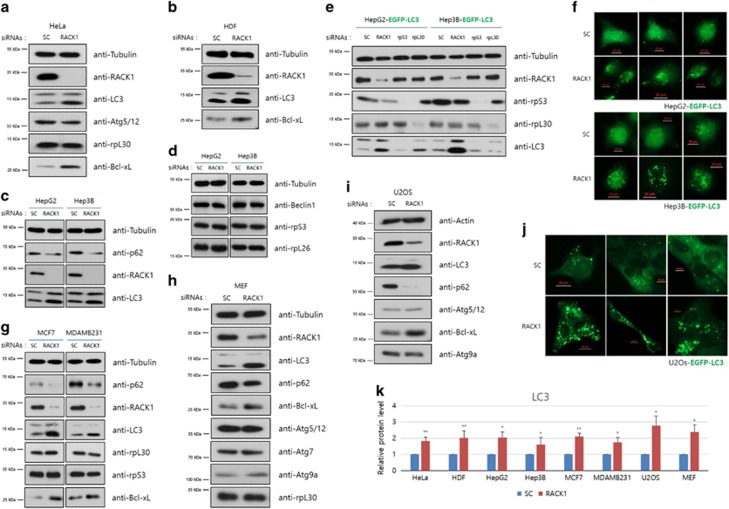
RACK1 depletion-induced autophagy is not a cell-line-specific phenomenon. (**a**–**d** and **g**–**i**) Control or RACK1 siRNAs were transfected (20 pmol) into HeLa (**a**), HDF (**b**), HepG2 and Hep3B (**c**, **d**), MCF7 and MDA-MB231 (**g**), MEF (**h**) and U2OS (**i**) cells. After 48 h, immunoblot analysis was performed using the indicated antibodies. (**e**, **f** and **j**) Indicated siRNAs (50 pmol) were transfected into EGFP-LC3 stably expressing HepG2, Hep3B and U2OS cells, followed by fluorescence microscopy (**f** and **j**) and immunoblot analyses using the indicated antibodies (**e**). (**k**) In each cell line, the intensities of LC3 proteins were normalized against that of tubulin protein, and the relative expression in RACK1 siRNA-treated cells compared with that in control cells was plotted. *P<0.05, **P<0.01 (Student’s *t*-test)

Thus far, we investigated the autophaic flux induced by RACK1-knockdown in HT1080 cells. To determine whether other cell lines show a similar phenomenon, several other cells were used. Autophagy induction by RACK1 knockdown was also observed in HeLa and U2OS cells (Figures 4a and i–k). This result is in a good agreement with our previous data in HT1080 cells. However, the incubation time after siRNA treatment differed between those cell lines (Supplementary Figures 1B and 3A). Moreover, this phenomenon was also detected in normal human dermal fibroblasts (HDFs) (Figures 4b and k and Supplementary Figure 3B). Similar to HeLa and U2OS cells, HDF cells also appeared to have an effective time for autophagy induction after RACK1 siRNA treatment (Supplementary Figure 3C). In two other HCC cell lines, HepG2 (p53 wild type) and Hep3B (p53 null) cells, the autophagy induction was also observed (Figures 4c and k). In addition, there was no noticeable increment in Beclin1 and decrement in several ribosomal proteins (Figure 4d). Indeed, using a fluorescence microscope, EGFP-LC3 stably expressing HepG2 and Hep3B cell lines also exhibited markedly increased numbers of GFP-LC3 puncta following RACK1 siRNA treatment (Figure 4f). Moreover, the LC3-II protein level was increased in RACK1-knockdown cells but not in rpS3- or rpL30-knockdown cells, and the fluorescence data showed identical patterns as well (Figure 4e and Supplementary Figure 3D). Besides, co-localization level of GFP-LC3 puncta and LysoTracker was also elevated in RACK1-knockdown cells (Supplementary Figure 3E). In conclusion, these results indicate that RACK1-knockdown-induced autophagy is neither a cell-line-specific nor a p53-dependent phenomenon. Furthermore, we proved that the phenomenon is not related to tumorigenic stages, using two breast cancer cell lines, MCF7 and MDA-MB231 cells (Figures 4g and k). In the breast cancer cell lines, non-canonical autophagy induction and Bcl-xL increment were also detected when RACK1 siRNA was treated. The similar phenomenon is observed in mouse embryonic fibroblasts (MEFs), in addition to several human cell lines (Figures 4h and k), and even observed in *Asc1*, a yeast RACK1 homolog, deletion strains in *Saccharomyces cerevisiae* (data not shown). To sum up, when cells were treated with RACK1 siRNA, LC3-II protein level is increased and p62 protein level is decreased, meaning autophagy induction, and Bcl-xL is also increased.

### Increment of LC3 and Bcl-xL occurs at the translational level

Our findings presented so far indicate that RACK1 knockdown induces non-canonical autophagy, accompanied by an increment in the level of LC3 and Bcl-xL proteins. Then, we were curious about how the level of those proteins was increased in RACK1-knockdown cells. RACK1 is a component of the 40S ribosomal subunit and may contribute to protein translation as a link between extracellular signals and the ribosome complexes. Additionally, RACK1 regulates the selective translation of viral IRES-containing mRNAs,^17^ and recruits the miRNA-induced silencing complex to the translating ribosome.^43^ Beclin1-independent autophagy, meanwhile, is related to IRES-dependent translation.^44^ Accordingly, to clarify the inductions of both LC3 and Bcl-xL in RACK1 depletion condition, we intended to examine the effect of ribosomal RACK1 depletion on mRNA translation. As depicted in Supplementary Figures S4A and B, ribosome fractionation of RACK1 siRNA-transfected cells were performed, followed by isolation of polysomes.^45^ The level of polysomal fraction peaks in RACK1-knockdown cells was decreased compared with control cells and the rate of protein synthesis was also slightly reduced (Supplementary Figure S4C and D). Subsequently, translating mRNA was extracted from these polysomal fractions, and the mRNA was analyzed (Supplementary Figure S4E).

**Figure 5:**
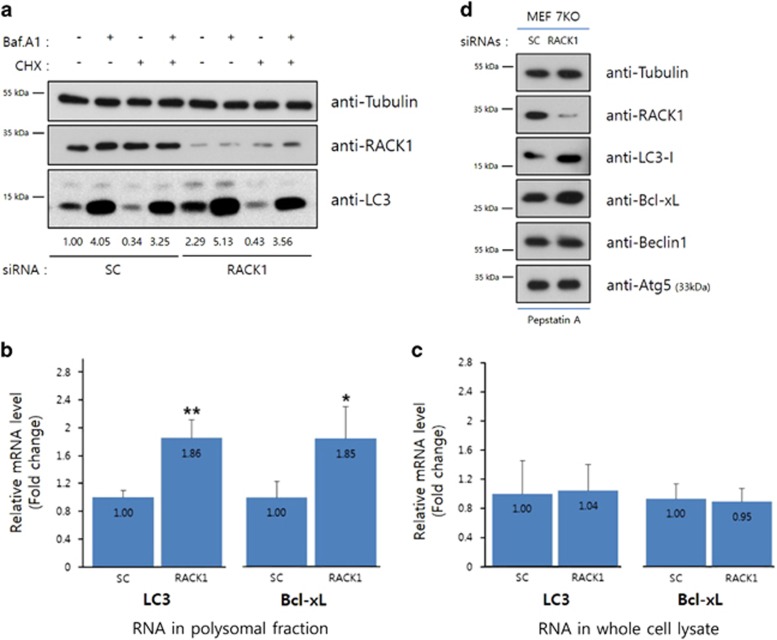
Increment of LC3 and Bcl-xL occurs at the translational level. (**a**) Control or RACK1 siRNA-transfected cells were incubated with 1 *μ*M Bafilomycin A1 and 50 *μ*g/ml cycloheximide for 1 h. The cell lysates were subjected to immunoblot analysis using the indicated antibodies. The numbers represent signal intensities normalized to the LC3-II level (first lane). (**b**) Relative LC3 and Bcl-xL mRNA levels in whole-cell lysates by real-time PCR. (**c**) Relative LC3 and Bcl-xL mRNA levels in polysomal fractions by real-time PCR. The mRNA levels were normalized to that of *β*-actin, and relative mRNA quantities divided by those in control cells are shown. *P<0.05, **P<0.01 (Student’s *t*-test). Data are representative of three independent experiments. (**d**) Control or RACK1 siRNAs were transfected (20 pmol) into ATG7 knockout MEF cells. Extracts of siRNA-transfected cells were pretreated with pepstatin A (2 ug/ml) for 16 h, and subjected to immunoblot analysis using the indicated antibodies

Based on our results and other previous reports, we suggested that ribosomal RACK1 depletion affects the repertoire of mRNA in actively translating ribosomes. To explore whether the increment in LC3 protein induced by RACK1 knockdown occurs at the translational level, cells were treated with a combination of cycloheximide and Bafilomycin A1 after treatment with several siRNAs. RACK1 knockdown increased the LC3 protein level compared with control (Figure 5a, lanes 1 and 5), and the increment was diminished to the basal level by cycloheximide treatment (Figure 5a, lanes 3 and 7). Co-treatment of cells with cycloheximide and Bafilomycin A1 induced a slight increase in the LC3 protein level following RACK1 knockdown comparison with control cells (Figure 5a, lanes 4 and 8). In summary, the increment of LC3 induced by RACK1 knockdown is regulated at the translational level, even if Bafilomycin A1 is considered as an inhibitor of the autophagosomal degradation of LC3 protein.

We next performed a real-time PCR assay of LC3 and Bcl-xL mRNAs in polysomes of RACK1-depleted cells. The real-time PCR data, which were normalized to the appropriate mRNA level of *β*-actin, indicated a significant increment in the translating LC3 and Bcl-xL mRNA levels in the polysomal fraction of RACK1-depleted cells compared with control cells (Figure 5b), while the levels of these mRNAs in whole-cell lysates were unchanged (Figure 5c). We performed the same experiment using a different LC3 primers and obtained a similar result (Supplementary Figure S5A). Therefore, in RACK1-knockdown cells, the increment in LC3 and Bcl-xL protein levels is caused by specific translation of the RACK1-depleted ribosome. To completely exclude the probability of canonical autophagy induced by RACK1 knockdown, we used Atg7 knockout MEF cells (Supplementary Figure S5B). Because Atg7 is an E1-like activating enzyme related to LC3 activation,^46^ knockout of Atg7 cannot induce the conversion of LC3-I into LC3-II that indicates the blockade of canonical autophagy. As shown in Figure 5d, in Atg7 knockout MEF cells, the increment of LC3-I and Bcl-xL protein levels by RACK1 siRNA treatment was also observed when treated with pepstatin A, which blocks the degradation of autophagolysosome (Figure 5d). Collectively, the results suggested that RACK1 depletion-induced autophagy is independent of Atg7, indicating non-canonical autophagy due to specific translation by the RACK1-depleted ribosome.

### Overexpression of RACK1^R36D/K38E^ also induces autophagy

**Figure 6:**
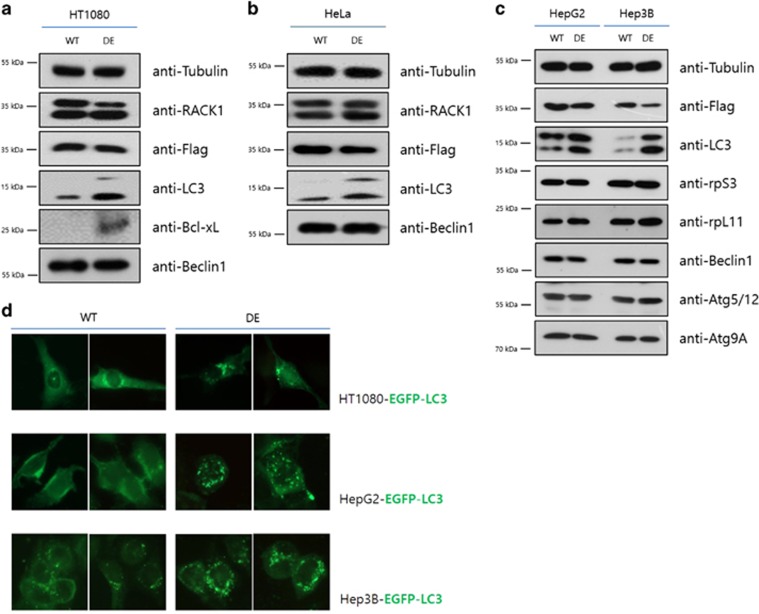
Overexpression of RACK1^R36D/K38E^ also induces autophagy. HT1080 (**a**), HeLa (**b**), HepG2 and Hep3B (**c**) cells were transfected with pcDNA3-FLAG-RACK and RACK1^R36D/K38E^. After 2 days, the levels of various proteins were assessed by immunoblot analysis. (**d**) pcDNA-FLAG-RACK1 and pcDNA-FLAG-RACK1^R36D/K38E^ were transfected into EGFP-LC3 stably expressing HT1080, HepG2 and Hep3B cells, and images were obtained by florescence microscopy

To determine whether depletion of ribosomal RACK1 influenced other canonical autophagic proteins, we transfected a plasmid of ribosome-binding defective mutant, RACK1 R36D/K38E (terms RACK1 DE mutant) plasmid into cells. The RACK1 DE mutant did not associate with polysomes (Supplementary Figures S6A and B) and the rate of protein synthesis was slightly reduced (Supplementary Figure S6C).^47^ In HT1080 cells, transfection of the RACK1 DE mutant increased the levels of LC-II and Bcl-xL proteins compared with RACK1 WT transfection (Figure 6a). Moreover, HeLa, HepG2 and Hep3B cells yielded similar results (Figures 6b and c). In addition, autophagosome formation was increased by transfection of RACK1 DE mutant. The number of GFP dots was greater in the RACK1 DE mutant-transfected cells than RACK1 WT-transfected cells, using three different cell lines stably expressing GFP-LC3 (Figure 6d). And the co-localization level of GFP-LC3 puncta and LysoTracker was also elevated in RACK1 DE mutant (Supplementary Figure S6D). Therefore, depletion of ribosomal RACK1 enhances autophagic flux, including increments in LC3 and Bcl-xL protein levels. Thus, the RACK1 status of a ribosome complex is a critical factor for non-canonical autophagy induction by LC3 mRNA-specific translation.

## Discussion

Autophagy is induced by various cellular stresses, such as starvation, hypoxia and viral infection. Under stress conditions, cells use autophagy to maintain the cellular homeostasis for the optimal levels of intracellular macromolecules and ATP. Non-canonical pathways of autophagosomal degradation by bypassing the canonical pathway have been identified.^36^ In case of emergency, most of the translational activities are decreased except stress-related proteins. The non-canonical autophagy may enable cells to cope with cellular emergencies promptly and efficiently. Here, we found that RACK1 knockdown induces non-canonical autophagic flux. We confirmed induction of events downstream of LC3, including LC3 puncta, increments in LAMP1 and LAMP2 protein levels, and degradation of p62/SQSTM1. Furthermore, with Atg7 knockout MEF, we clearly demonstrated that RACK1-induced autophagy is activated via an Atg7 independent manner (Figure 5d). These data suggest that ribosomal RACK1 depletion promotes non-canonical autophagy that is dependent on LC3, but differs from canonical autophagy, which requires Ulk1, conjugated Atg5/12, Atg7 and Beclin1. RACK1 knockdown also increased the level of Bcl-xL protein (Figure 3a), followed by a significant increase in the Bcl-xL–Beclin1 interaction, indicating the repression of canonical autophagy (Figures 2f and 3c).

It has been recently known that arsenite induces autophagy.^48, 49, 50, 51^ Moreover, Smith *et al.*^52^ suggested that arsenic trioxide induces non-canonical autophagy. Arimoto *et al.*^47^ also showed that RACK1 is recruited into stress granules under stress conditions. Moreover, LC3-associated non-canonical autophagy has been discovered to promote immune responses through activation of antigen presentation and control of interferon production, suggesting that non-canonical autophagy might play a crucial role in the response to extracellular stimuli.^53^ Therefore, we supposed that RACK1 depletion-induced non-canonical autophagy might also play a role in the physiological circumstances.

Emerging evidence suggests that ribosome heterogeneity resulting from the alteration of ribosomal components regulates the selective translation of specific mRNAs.^54^ Similarly, ribosomal RACK1 contributes to the selective translation of specific mRNAs,^55^ and is involved in IRES-dependent translation after viral infection stress.^17^ Here, as global translation and polysomal fraction were slightly decreased in RACK1 siRNA-transfected cells (Supplementary Figures S4C and D), depletion of ribosomal RACK1 affects the translation of specific mRNAs, such as LC3 and Bcl-xL (Figure 5b). Thus, in RACK1 siRNA-transfected cells, translation of specific mRNAs is dependent on the presence of ribosomal RACK1 in the ribosome complex.

We differentiated the role of ribosomal RACK1 from that of non-ribosomal or cytoplasmic RACK1. RACK1 plays various roles in the cytoplasm. However, because RACK1 has no catalytic domain,^3^ its roles vary according to its cellular localization and binding partners. Consequently, RACK1 could have diverse effects on physiological phenomena, depending on the cellular context. For example, RACK1 can promote tumorigenicity of non-small-cell lung cancer through hedgehog signaling,^56^ and the proliferation, invasion and metastasis of prostate cancer.^57^ In contrast, a lack of RACK1 could induce metastasis of gastric cancer through miRNA-mediated mechanisms.^58^ Although we found that ribosomal RACK1 depletion induces non-canonical autophagy, it is possible that RACK1 contributes to other steps in autophagic flux due to its interactions with diverse binding partners. In fact, RACK1 is related to the upstream signaling pathway of canonical autophagic flux.^21, 59^ On the contrary to these previous studies, our results indicate that loss of ribosomal RACK1 induces non-canonical autophagic flux increased the downstream event of LC3. Using the RACK1 DE mutant, we confirmed that our findings were due to depletion of ribosomal RACK1 but not non-ribosomal RACK1, which is not associated with the ribosome complex, as shown in Figure 6. However, we hypothesize that non-ribosomal RACK1 disappeared earlier than ribosomal RACK1 during RACK1 siRNA treatment. Indeed, RACK1 depletion-induced autophagy was dependent on the siRNA concentration and the incubation time after siRNA treatment (Supplementary Figures S1B and C).

## Materials and Methods

### Cell culture, antibodies and reagents

The human fibrosarcoma cell line HT1080, hepatocellular carcinoma cell lines HepG2 and Hep3B, cervical cancer cell line HeLa, bone osteosarcoma cell line U2OS, breast cancer cell lines MCF7 and MDA-MB231 and HDFs were obtained from the Korean Cell Line Bank (Seoul, Korea). MEF was obtained from the Yonsei University. These cell lines were cultured in DMEM/high glucose medium (Hyclone, GE Healthcare Life Science Korea, Seoul, Korea, SH30243.01) supplemented with 10% fetal bovine serum (FBS, Hyclone) and antibiotic–antimycotic (Gibco, ThermoFisher Scientific, Waltham, MA, USA, 15240-062). Antibodies against RACK1, rpL30, p62/SQSTM1, Atg7, Atg12 and Beclin1 were purchased from Santa Cruz Biotechnology (Dallas, TX, USA), antibodies against phosphor-rpS6, phospho-Ulk1, rpL26, LC3, Bcl-2, Bcl-xL, FLAG and *α*-tubulin were obtained from Cell Signaling Technology (Danvers, MA, USA). Antibodies against Atg5, Atg9A, Atg4B, Atg16, LAMP1 and LAMP2 were obtained from ProSci (Poway, CA, USA), and the anti-rpS3 antibody was from HAEL (Seoul, Korea). HRP-conjugated goat anti-rabbit and goat anti-mouse secondary antibodies were purchased from Jackson ImmunoResearch (West Groove, PA, USA). To induce cell starvation, HBSS (Gibco, 14025-092) was used. Bafilomycin.A1, an autophagy inhibitor, was from AG Scientific and cycloheximide, a translation inhibitor, was from Sigma-Aldrich (St. Louis, MO, USA). Chemiluminescence blotting substrate for immunoblot analysis was purchased from Boehringer Mannheim (Mannheim, Germany) and Lipofectamine 2000 and Lipofectamine RNAiMAX were purchased from Invitrogen (Carlsbad, CA, USA).

### Transfection of siRNA or plasmid

For siRNA transfection, specific siRNAs for RACK1 (sc-36354), rpL30 (sc-77598), ATG5 (sc-41445) and Beclin1 (sc-29798) were purchased from Santa Cruz. Scrambled control (SN-1013), RACK1 (1063081) and RACK1-3 (1063083) siRNAs were purchased from Bioneer (Daejeon, Korea). RpS3 and RACK1 5′-UTR siRNAs were constructed by GE Healthcare Dharmacon (Lafayette, CO, USA). The above siRNAs were reverse transfected using Lipofectamine RNAiMAX according to the manufacturer’s recommendations. For pEGFP-LC3, pcDNA3-FLAG-RACK and RACK1^R36D/K38E^ transfection, the Lipofectamine 2000 reagent (Invitrogen) was used.

### Immunoblot assay

Cells were harvested and lysed with lysis buffer (Tris-Cl (pH 7.5), 150 mM NaCl, 2.5 mM MgCl_2,_ 1 % Triton X-100, 0.25 % Na-deoxycholate, 2 mM PMSF, 1 *μ*g/ml aprotinin, 1 *μ*g/ml leupeptin) for 30 min on ice. The supernatants were collected by centrifugation at 12 000 × *g* for 10 min at 4 °C, and protein concentrations were determined using the Bradford reagent (Bio-Rad, Hercules, CA, USA, #500-0006). Whole-protein lysates were boiled in 2 × SDS-PAGE sample buffer, separated by SDS-PAGE, and transferred to an NC or PVDF membrane for immunoblotting.

### Immunoprecipitation

Cells were harvested and lysed in lysis buffer for 30 min on ice. The supernatants were collected by centrifugation at 12 000 × *g* for 10 min at 4 °C. Whole-cell lysate (1 mg) was incubated with 2 *μ*g of specific primary antibodies for >2 h at 4 °C, and the immunoprecipitates were collected using protein A- or G-agarose beads. After extensive washing with lysis buffer, the immunoprecipitates were resolved on an SDS-PAGE gel and subjected to immunoblot analysis.

### Ribosomal fractionation and ribosomal pelleting

Cells were harvested after adding cycloheximide (100 *μ*g/ml) to the medium for 30 min, and lysed with 1 ml of lysis buffer (Tris-Cl (pH 7.5), 150 mM NaCl, 2.5 mM MgCl_2,_ 1% Triton X-100, 0.25% Na-deoxycholate, 2 mM PMSF, 1 mg/ml aprotinin, 1 mg/ml leupeptin, 1 mM DTT, 100 units/ml RNasin (Promega, Madison, WI, USA, N251B), 50 *μ*g/ml CHX). The cell lysates were then applied to a sucrose gradient or sucrose cushion. The sucrose gradient buffer comprised 20 mM Tris-Cl (pH 7.5), 150 mM NaCl, 2.5 mM MgCl_2_ with DEPC (Sigma, D5758) DW. The cell lysates were subjected to ultracentrifugation at 32 000 r.p.m. for 3 h 10 min at 4 °C in a Beckman SW41Ti rotor (Brea, CA, USA).

### Reverse transcription-polymerase chain reaction (RT-PCR) and real-time PCR

Total cellular RNA was extracted using TRIzol reagent (Ambion, 15596-018). Translating mRNA from the ribosomal fraction was extracted using the TRIZOL LS reagent (Ambion, ThermoFisher Scientific, Waltham, MA, USA, 10296-028) after ribosomal pelleting. These RNAs were reverse-transcribed using oligo-dT_15_ primers (Promega) and M-MLV reverse transcriptase (Promega, M170A). Subsequently, gene expression and translation were quantified by real-time PCR of the above-synthesized cDNAs, using a Light Cycler 480 instrument (Roche Molecular Biochemicals, Basel, Switzerland). The sequences of primers used for real-time PCR analysis were as follows. LC3, 5′-CCA CAC CCAAAG TCC TCA CT-3′ and 5′-CAC TGC TGC TTT CCG TAA CA-3′ LC3-2, 5′-GAG AAG CAG CTT CCT GTT CTG G-3′ and 5′-GTG TCC GTT CAC CAA CAG GAA G-3′ Bcl-xL, 5′-GAT CCC CAT GGC AGC AGT AAA GCA AG-3′ and 5′-CCCCAT CCC GGA AGA GTT CAT TCA CT-3′ beta actin, 5′-GTA CCA CTGGCA TCG TGA TGG ACT-3′ and 5′-CCG CTC ATT GCC AAT GGT GAT-3′ 18S rRNA, 5′-GTAACCCGTTGAACCCCATT-3′ and 5′-CCATCCAATCGGTAGTAGCG-3′. The significance of differences was determined by Student’s *t*-test.

### Microscopy

After treatment with siRNAs, HT1080-EGCP-LC3 cells were visualized using a fluorescence microscope (Carl Zeiss, Oberkochen, Germany, Axioscope). The cells were washed twice with PBS and fixed in paraformaldehyde (PFA) for 15 min. To observe lysosomes, the cells were incubated with 50–75 nM LysoTracker Red DND-99 (Thermo Fisher Scientific, L-7528) for 30 min at 37 °C. The number of autophagosome per cell and the size of autophagsome are measured by Operetta High-Content Imaging System and Harmony High-Content Imaging and Analysis Software (PerkinElmer, Waltham, MA, USA, HH12000000 and HH12000701), using Cyto-ID Autophagy Detection Kit (Enzo LifeScience, Farmingdale, NY, USA, ENZ-51031).
