# Supplementary material for: RACK1 depletion in the ribosome induces selective translation for non-canonical autophagy
Source: Cell Death Dis. 2017 May 18;8(5):e2800–. doi: 10.1038/cddis.2017.204 (PMC5520723; doi:10.1038/cddis.2017.204)
Supplement: Supplementary Figures [file cddis2017204x1.ppt]

## Slide 1
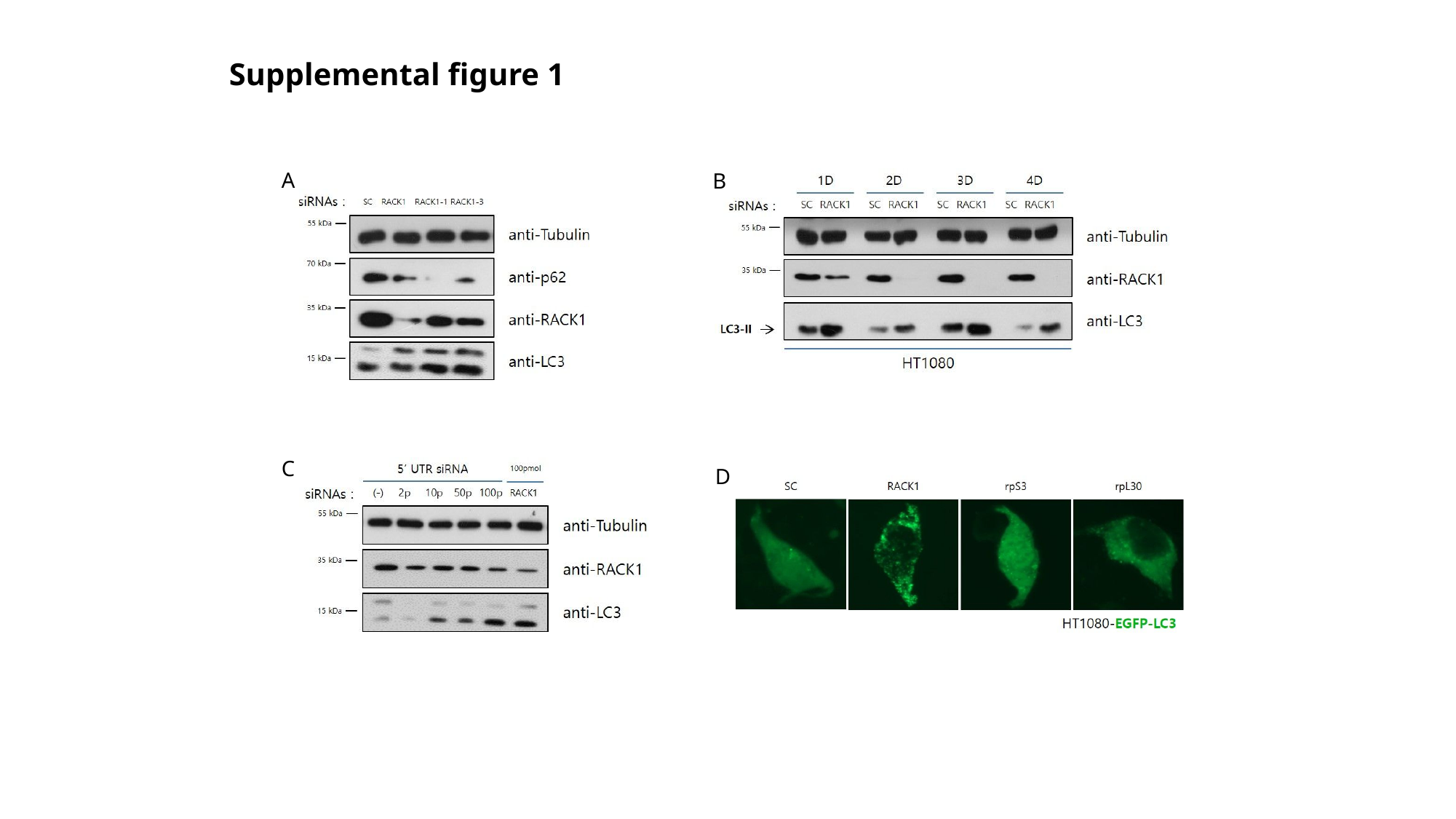

Supplemental figure 1
A
B
C
D

## Slide 2
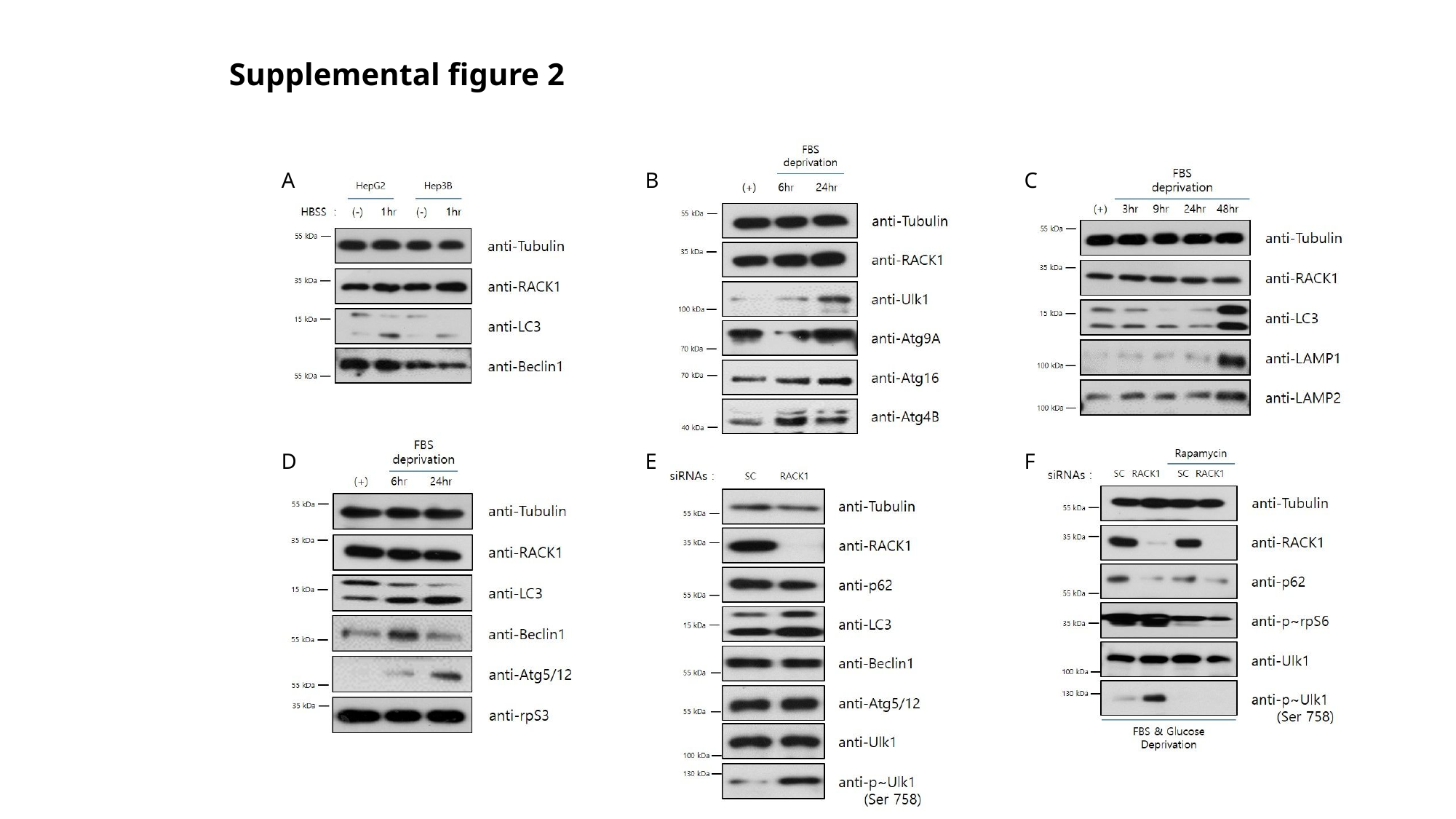

Supplemental figure 2
A
B
C
D
E
F

## Slide 3
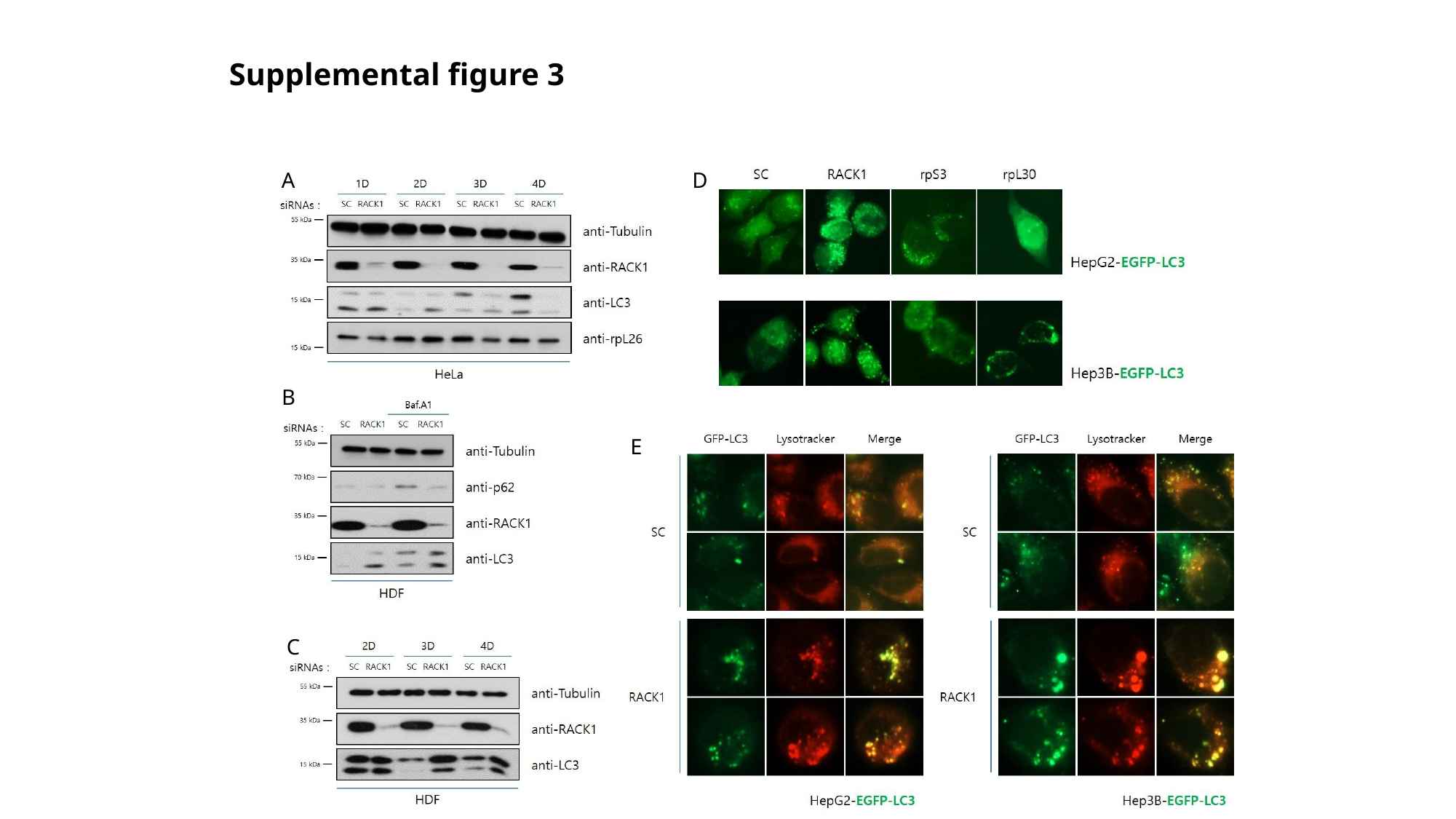

Supplemental figure 3
A
D
B
E
C

## Slide 4
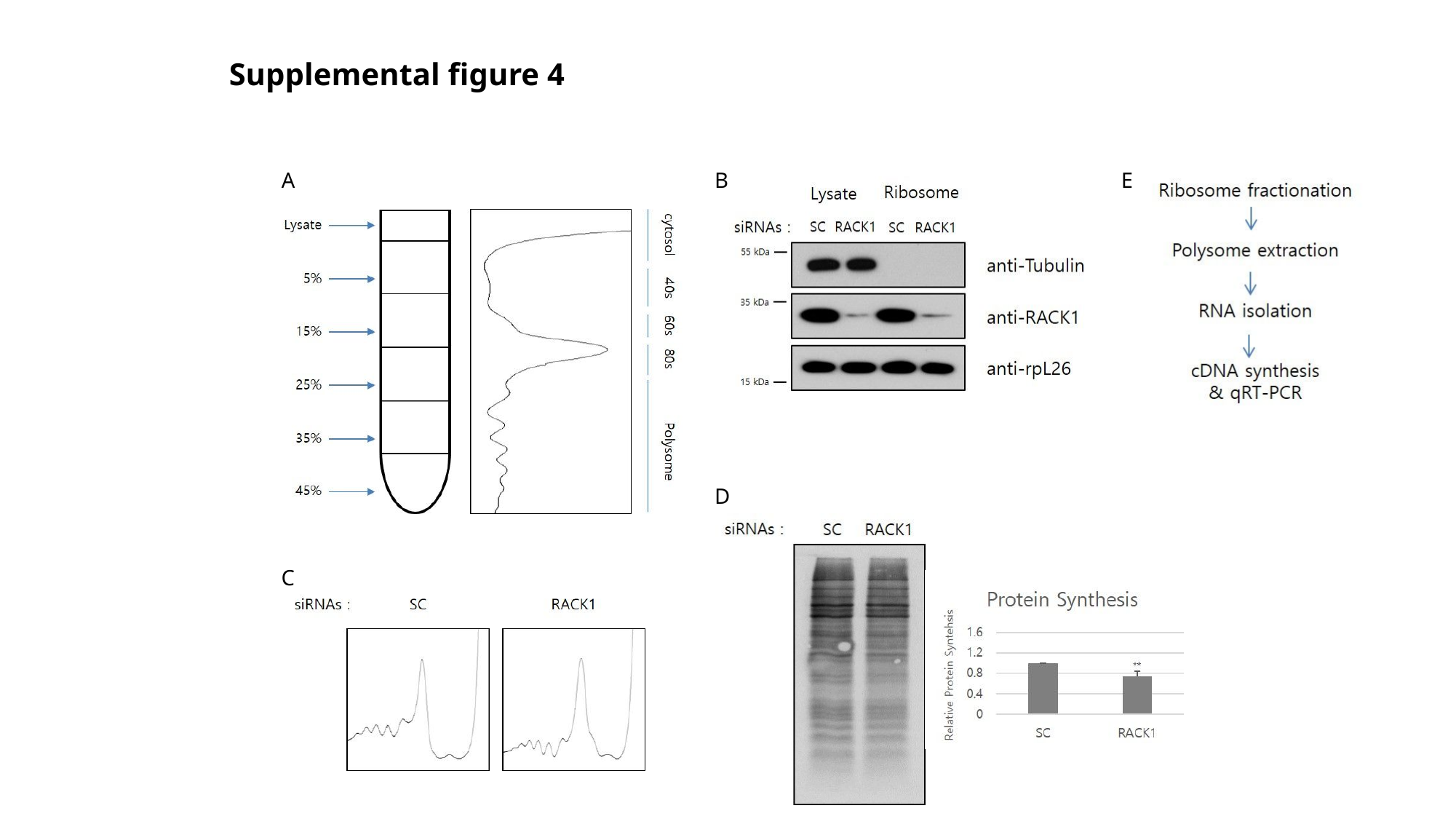

Supplemental figure 4
A
E
B
D
C

## Slide 5
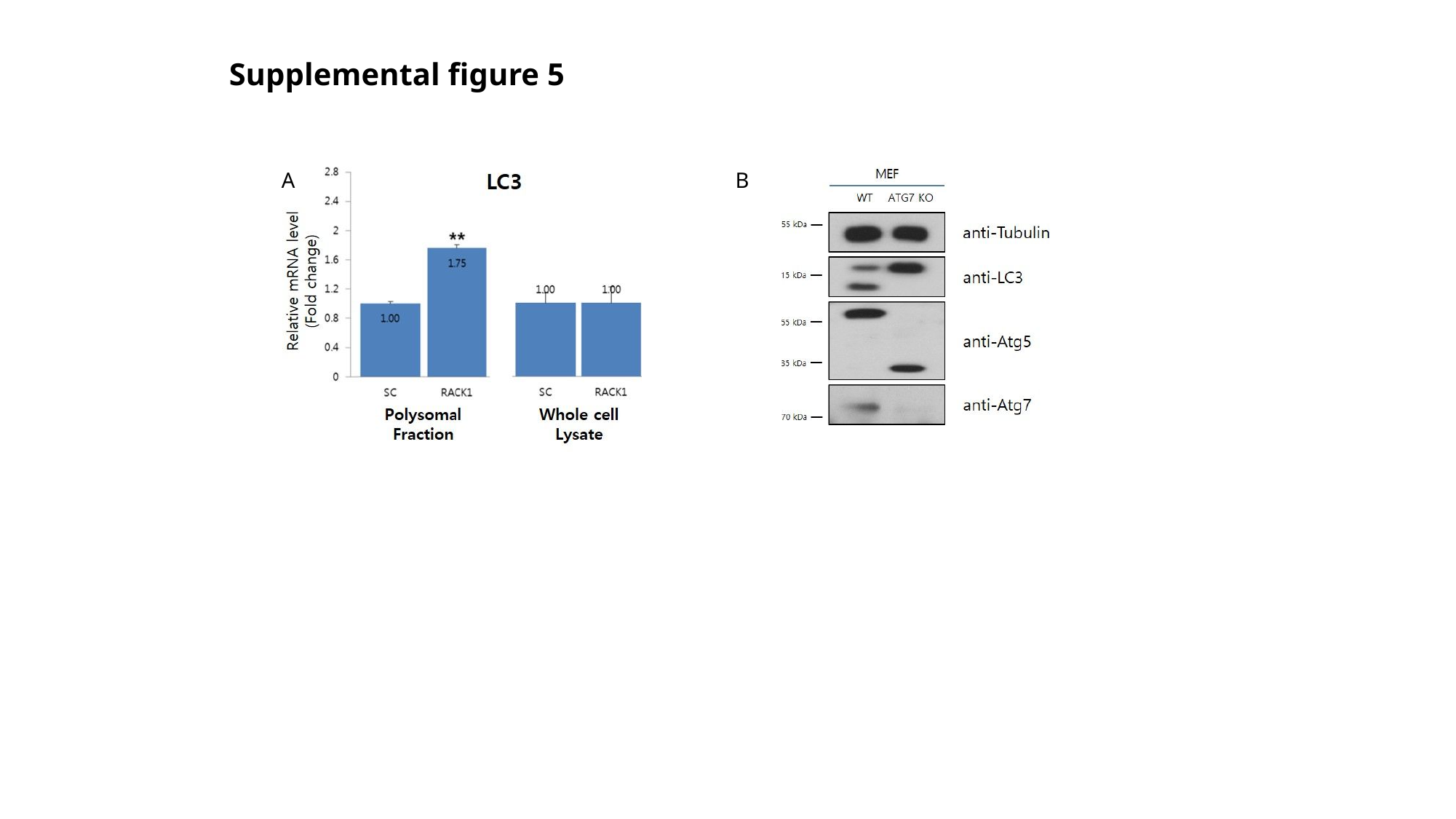

Supplemental figure 5
A
B

## Slide 6
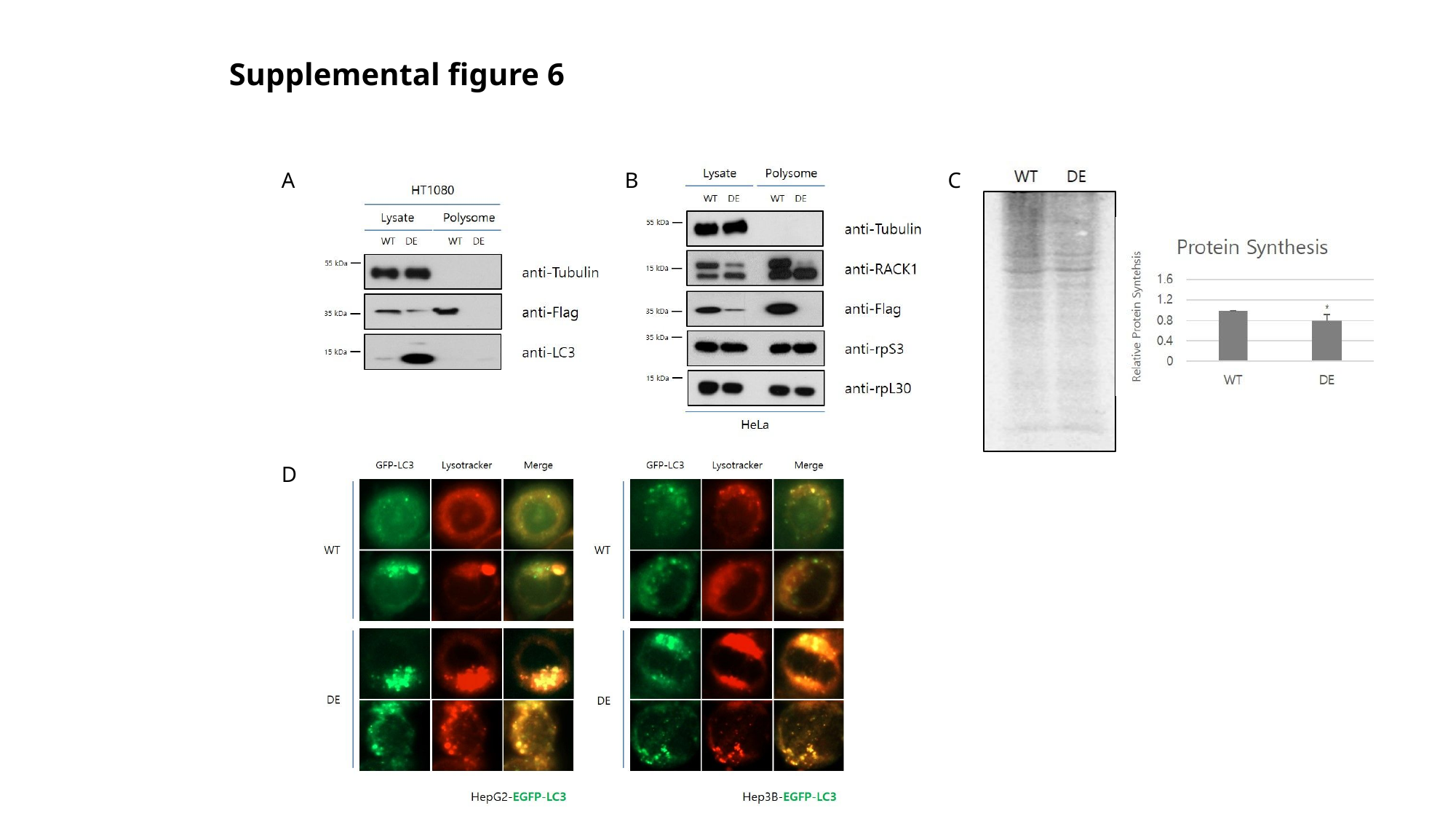

Supplemental figure 6
A
B
C
D
