# Supplementary material for: RACK1 depletion in the ribosome induces selective translation for non-canonical autophagy
Source: Cell Death Dis. 2017 May 18;8(5):e2800–. doi: 10.1038/cddis.2017.204 (PMC5520723; doi:10.1038/cddis.2017.204)
Supplement: Supplementary Figure Legends [file cddis2017204x2.docx]

**Supplemental Figure 1.**

(A-C) Immunoblot analyses using the indicated antibodies were performed after treatment of siRNAs for control and RACK1 in HT1080 cells. (A) HT1080 cells were transfected with three different siRNAs for RACK1 (100 pmol). RACK1 was the siRNA used in this article; the other siRNAs target different RACK1 mRNA sequences. (B) After treatment with control and RACK1 siRNAs (20 pmol), HT1080 cells were harvested after 1–4 days. (C) HT1080 cells were treated with 2–100 pmol RACK1 siRNA, which targets the 5’ UTR sequence. RACK1 was the siRNA used in this article. (D) Indicated siRNAs (50pmol) were transfected into EGFP-LC3 stable HT1080 cells. GFP-LC3 dots were detected by fluorescence microscopy.

**Supplemental Figure 2**.

(A) HT1080 cells were cultured in HBSS for 1 h, harvested, lysed, and analyzed by immunoblotting using the indicated antibodies. (B-D) HT1080 cells were serum-starved for the indicated time periods, and the cell lysates were subjected to immunoblot analysis using the indicated antibodies. (E) HT1080 cells were transfected with control or RACK1 siRNAs (50 pmol), and then incubated for 48 h. The cell lysates were subjected to immunoblot analysis using the indicated antibodies. (F) HT1080 cells were transfected with control or RACK1 siRNAs (50 pmol), and then incubated for 48 h. The cells were incubated in glucose free DMEM without serum for 1 hour and pretreated with rapamycin 1 uM for 4 h in the same media.

**Supplemental Figure 3**.

After treatment with control and RACK1 siRNAs (20 pmol), HeLa (A) and HDF (C) cells were harvested after 1–4 days. The cell lysates were then subjected to immunoblot analysis using the indicated antibodies. (B) Immunoblot analyses using the indicated antibodies were performed after treatment with control and RACK1 siRNAs (20 pmol) in HDF cells. The cell extracts from siRNA-transfected cells were pretreated with Bafilomycin A1 (1 µM) for 1 h. (D) Indicated siRNAs (50 pmol) were transfected into EGFP-LC3 stably expressing HepG2 and Hep3B cells, followed by fluorescence microscopy. (E) Control or RACK1 siRNAs (50 pmol) were transfected into EGFP-LC3 stably expressing HepG2 and Hep3B cells. After 48 h, these cells were incubated with 50 nM LysoTracker for 30 min. Cells were then washed with PBS, and images obtained by florescence microscopy.

**Supplemental Figure 4**.

(A) Schematic diagram of ribosome fractionation by sucrose gradient ultracentrifugation. Sucrose gradients of 5–45 % were used. Peaks for 80S, 60S, 40S and several polysomal peaks are observed. (B) HT1080 cells were treated with control or RACK1 siRNAs (50 pmol). After 48 h, the ribosomal peak patterns were analyzed by sucrose gradient fractionation. (C) The above cell lysates and ribosomal fractions were subjected to immunoblot analysis with the indicated antibodies. RpS3 was used as a marker of the ribosomal fraction and tubulin of the non-ribosomal fraction. (D) HT1080 cells were treated with control or RACK1 siRNAs (50 pmol). After 48 h, the cells were subjected to metabolic labeling with [^35^S]-methionine followed by incubation for 30 min. Labeled cell lysates were resolved by SDS-PAGE and subsequently analyzed by a phosphorimager. (E) Translational mRNA analysis procedure. More than 1 mg of cell lysates were used for ribosome fractionation.

**Supplemental figure 5**.

(A) LC3 mRNA level in whole cell lysates (left panel) and the polysomal fractions (right panel), analyzed by real-time PCR. mRNA levels were normalized to that of β-actin, and relative mRNA quantities divided by those in control cells are shown. *; p<0.05, **; p<0.01 (Student’s t-test). The data are representative of three independent experiments. (B) Wild type MEF and ATG7 knockout MEF cells were subjected to immunoblot analysis using the indicated antibodies.

**Supplemental figure 6**.

(A, and B) After 48 h from transient transfection with pcDNA3-Flag-RACK1 and pcDNA3-FLAG-RACK1^R36D/K38E^, immunoblot analyses using the indicated antibodies were separately performed on the polysomal fraction and whole cell lysate of HT1080 (A), and HeLa (B) cells. (C) After 48 h from transient transfection with pcDNA3-Flag-RACK1 and pcDNA3-FLAG-RACK1^R36D/K38E^ into HT1080, the cells were subjected to metabolic labeling with [^35^S]-methionine followed by incubation for 30 min. Labeled cell lysates were resolved by SDS-PAGE and subsequently analyzed by a phosphorimager. (D) After transfection with pcDNA-Flag-RACK1 and pcDNA-FLAG-RACK1^R36D/K38E^ into GFP stably expressing HepG2 and Hep3B cells, cells were incubated with 50 nM LysoTracker for 30 min. Using a fluorescence microscope, the co-localization of lysosomes (red LysoTracker) and autophagosomes (green GFP-LC3) appeared as a yellow color, which indicates an autophagolysosome.
